# Mental Health Problems and Associated Factors among High School Students in Shandong Province of China: A Cross-Sectional Study

**DOI:** 10.3390/ijerph19148478

**Published:** 2022-07-11

**Authors:** Xiaolei Xiu, Qing Qian, Sizhu Wu

**Affiliations:** Department of Medical Data Sharing, Institute of Medical Information/Library, Chinese Academy of Medical Sciences & Peking Union Medical College, 3 Yabao Road, Chaoyang District, Beijing 100020, China; xiu.xiaolei@imicams.ac.cn (X.X.); qian.qing@imicams.ac.cn (Q.Q.)

**Keywords:** high school student, parents’ educational level, physical activity, school adaptation, mental health problems, China

## Abstract

Background: Although many studies have analyzed the mental health problems (MHP) of Chinese adolescents, the associations of Chinese high school students’ MHP with personal and family circumstances, parents’ educational level, physical activity, and school adaptation are rarely discussed. Methods: The participants were 9398 students who were randomly recruited from 30 high schools in Shandong, China. Self-reported questionnaires were used to collect data. Multivariate logistic regression models were used to investigate associations between MHP and related factors. Results: The positive rate of MHP among high school students was 27.0%. Female, intimate friends of the opposite sex, poor family economic status, father’s educational level of primary school or below, low social competence, and high antisocial behavior were significantly associated with higher odds of having MHP (*p* < 0.05). Students’ self-reported odds of having obsessive–compulsive disorder, interpersonal sensitivity, and depression were inversely related to their mother’s educational level. Compared with students who did not exercise, students who engaged in more than 30 min of physical activity 5–6 times per week had 60% lower self-reported odds of depression. Conclusions: We recommend strengthening the prevention, early detection, and treatment of MHP in high school students, especially those whose parents have low educational attainment, inactivity, and high disruptive behaviors.

## 1. Introduction

Mental health problems (MHP) have become a major disease burden among Chinese adolescents [1]. It is estimated that at least 30 million children and adolescents under the age of 17 in China have experienced emotional or behavioral problems [2]. Poor mental health in adolescents is associated with serious long-term consequences, such as substance abuse, lower educational attainment, violence, self-harm, and even suicide, which may persist even into adulthood [3]. Adolescents in high school are a very special group. Unlike junior high school students, high school students faced many new challenges, including adjusting to a new environment, making new friends, academic challenges, and National College Entrance Examination (NCEE), which makes them more vulnerable to MHP. Studies have shown that half of MHP begin by the age of 14, and most cases go undetected and untreated [4]. In addition, according to the “Report on National Mental Health Development in China (2019–2020)”, the detection rate of depression in primary school is about 10%, while that in high schools reaches 40%. Therefore, it is crucial to strengthen the early detection and prevention of MHP in high school students.

Mental health problems are the product of the interaction of many factors. During adolescence, these influencing factors may include personal factors, family factors, peer relationships, and broader circumstances such as school adaptation. Existing studies have explored the influence of various factors such as gender, place of residence, single and non-single child, and socioeconomic status on adolescent mental health, as well as the correlation between physical activity (PA) and mental health [5,6,7,8]. The findings suggest that there is a positive correlation between PA and mental health and that enhancing PA can reduce MHP in adolescents [9,10]. However, with regard to the frequency or intensity of PA, most studies only classify it into high, moderate, and low levels based on activity type and activity duration [11,12]. This coarse-grained classification is not conducive to formulating specific psychological interventions. Furthermore, Bell et al. found that PA has the potential to reduce depression and anxiety symptoms in adolescents, but they found no strong evidence that PA was associated with better mental health or reduced symptoms of mental health disorders [13]. Therefore, it is necessary to conduct a more detailed grading of PA, and comprehensively discuss the relationship between PA and various MHP, such as obsessive–compulsive disorder, interpersonal sensitivity, and anxiety.

Parents, as the shapers of children’s psychological world, their educational attainment has a significant impact on adolescents’ mental health. Previous studies have found that parents’ educational level (PEL) is inversely related to their children’s MHP, that is, higher educational attainment of parents means less MHP in adolescents [14,15]. However, some studies indicated that the influence of PEL on adolescents’ mental health was entirely achieved through the adolescent’s own educational attainment. Additionally, the educational attainment of fathers and mothers had different effects on adolescents, for example, adolescents’ self-compassion was associated with the educational attainment of the father, but not the educational attainment of the mother [16,17]. The extent to which fathers’ and mothers’ educational attainment affects adolescents’ mental health remains to be discussed.

Chinese high school students spend most of their time at school, which is also recognized as one of the key environments for psychological interventions for adolescents [18,19]. Previous studies have investigated associations between school adaptation and adolescents’ mental health. For example, students with poor interpersonal relationships are vulnerable to somatization, impulsiveness, or depression [20]. Good teacher–student relationships are associated with better adolescents’ mental health [21]. However, school failures leave adolescent girls vulnerable to depression in young adulthood [22]. School adaptation refers to the series of efforts that students make to cope with the stress caused by learning, relationships with teachers, relationships with friends, and school life in order to live in harmony with school [23]. It can be seen that school adaptation is the result of the combined effect of various factors such as personal characteristics, and relationships with classmates and teachers. A comprehensive analysis of the correlation between various factors of school adaptation and MHP is more conducive to early intervention for students’ MHP.

In conclusion, PEL, PA, and social adaptation have crucial effects on adolescents’ mental health. Although prior studies have explored the association between PEL, PA, or social adaptation and adolescent MHP, there are several problems. First, most studies on MHP have been conducted on children or adolescents, a mixed sample including middle and high school students [8,24]. There are few studies on the MHP of Chinese high school students alone, especially analyzing the association between PEL, PA, school adaptation, and MHP among Chinese high school students. Second, existing studies on the correlation between PEL and MHP are controversial, and the relationship between PA frequency and MHP remains to be revealed. Moreover, school adaptation includes two complex dimensions of social competence and antisocial behavior [25]. At present, there are few studies on the relationship between high school students’ antisocial behavior and MHP. Third, MHP includes somatization, obsessive–compulsive disorder, interpersonal sensitivity, and other psychological symptoms. Most of the current papers focused on the relationship between associated factors and MHP [3,6]. Less research has been performed on hostility, phobic anxiety, and paranoid ideation.

There are 31 provincial-level administrative divisions in mainland China. According to 2020 data from the Ministry of Education of the People’s Republic of China, there were 41.278 million high school students nationwide, of which about 1/16 (2.5372 million) were from Shandong Province. There are a large number of candidates for the NCEE in Shandong Province, and the NCEE in Shandong Province is more difficult than in other provinces. High school students in Shandong Province are under enormous academic and competitive pressures. This study aims to comprehensively evaluate the current mental health status of high school students in Shandong Province and use the SCL-90 subscale to explore in detail the association between high school students’ MHP and PEL, PA, and school adaptation. Inspired by the findings of previous studies, we hypothesize that higher PA frequency, higher PEL, and better social competence would contribute to improving MHP, whereas high levels of antisocial behavior would worsen MHP. The results of this study will provide a theoretical basis for effective and specific psychological intervention for various psychological problems of Chinese high school students.

## 2. Materials and Methods

### 2.1. Participants and Study Design

This study is a cross-sectional study of students recruited from 30 high schools in 10 administrative districts of Shandong Province, China, during the 2020–2021 semester. According to the specific geographical, demographic, and socio-economic levels of each administrative district, 30 high schools were randomly selected using the probability-proportional-to-size sampling method [26]. Schools with fewer than 100 students per grade were excluded from this study. In addition, more than 300 participants were randomly selected from each high school using simple random sampling. Finally, 9480 questionnaires were returned. After excluding the questionnaires due to missing or illogical information in the data analysis, there were 9398 valid questionnaires. Teachers, parents, and students filled out a consent form prior to enrollment in this survey. The research has been approved by the Ethics Committee of Shandong University, China (20180517).

### 2.2. Data Availability

The data presented in this study are openly available in the Population Health Data Archive [27].

### 2.3. Measurements

#### 2.3.1. Demographic Information

Demographic factors: These include gender, place of residence, single-child household or not, having intimate friends of the opposite sex or not, in the top class or not, and family economic status. The two options for place of residence are urban and rural areas. The family’s economic status includes three choices: poor, middle, and good.

Parents’ educational level (PEL): In this study, fathers’ and mothers’ educational level is divided into 5 categories: elementary school and below, junior high school, high school, university, postgraduate and above.

#### 2.3.2. Physical Activity Measure

Physical activity (PA): PA was evaluated by the Physical Activity Questionnaire for Adolescents (PAQ-A). This scale is a revised version of the Physical Activity Questionnaire for Children (PAQ-C), which is designed to assess the PA level of adolescents [28]. It is on a 5-point scale (1–5), with higher scores indicating higher PA levels. Results can be divided into two categories: low PA levels (1–1.9 points) and high PA levels (2–5 points) [29]. The validity and reliability of PAQ-A have been validated in Chinese adolescents [30]. Furthermore, this paper investigated how many times over 30 min of PA high school students had completed in the past week, with options ranging from 0 times, 1–2 times, 3–4 times, 5–6 times, and ≥7 times. The Cronbach’s alpha and McDonald’s omega coefficients of the scale in this study were 0.82 and 0.87, respectively. In addition, exploratory principal component factor analysis (EFA) and confirmatory factor analysis (CFA) were also performed: Kaiser–Meyer–Olkin (KMO) = 0.83, Bartlett’s test *p* < 0.001, Average Variance Extracted (AVE) = 0.48, Composite Reliability (CR) = 0.82. The fit indices for this scale were at an acceptable level (X^2^/df = 55.78, GFI = 0.99, RMSEA = 0.076, CFI = 0.98, NFI = 0.98, TLI = 0.96, SRMR = 0.022). See Supplementary Table S1 for details.

#### 2.3.3. School Adaptation Measure

School adaptation: School adaptation was measured by the School Social Behavior Scale-2 (SSBS-2). SSBS-2 is a teacher rating for two composite dimensions of Social Competence (including three subscales: peer relations, self-management/compliance, and academic behavior) and antisocial behavior (including three subscales: hostile/irritable, antisocial/aggressive, and defiant/disruptive) [25]. It has 64 items rated on a 5-point Likert scale ranging from 1 (never) to 5 (often). SSBS-2 has good test-retest reliability, inter-evaluator agreement, high internal consistency, and fit indices in the confirmatory factor analysis [31]. In this study, Cronbach’s alpha ranged from 0.90 to 0.97 and McDonald’s omega ranged from 0.92 to 0.95. The EFA showed that the scale had good construct validity (KMO = 0.98, Bartlett’s test *p* < 0.001), and the CFA also showed good convergent validity and fit (Table S2).

#### 2.3.4. Mental Health Problem Measure

Mental health problems: Mental health problems were assessed by the Symptom Checklist-90 (SCL-90) scale for high school students, a self-reported mental health questionnaire designed to screen for a wide range of psychological problems [32]. The SCL-90 consists of 90 items rated on a 5-point Likert scale ranging from 1 (none) to 5 (severe) and includes the following 9 psychiatric symptom factors: somatization, obsessive–compulsive, interpersonal sensitivity, depression, anxiety, hostility, phobic anxiety, paranoid ideation, psychoticism. The Global Severity Index (GSI) is equal to the average of the nine subscale scores. A subscale score ≥3 is considered clinical positive with symptoms. Exploratory factor and correlation analysis in multiple studies have demonstrated satisfactory reliability and validity of the SCL-90 scale [33,34]. In this study, high internal consistency was demonstrated: Cronbach’s alpha ranged from 0.85 to 0.94 and McDonald’s omega ranged from 0.89 to 0.95. Moreover, we performed EFA (KMO = 0.99 and Bartlett’s test *p* < 0.001) and CFA (Table S3), and the results showed that the scale had an acceptable validity.

### 2.4. Statistical Analysis

The SCL-90 total score and its nine subscale scores were the main interest of this study. Continuous variables were expressed as mean and standard deviation, and categorical variables were expressed as numbers and percentages. Independent samples 2-tailed *t*-tests and one-way analysis of variance tests were used to compare the differences between groups of categorical variables, as appropriate. Furthermore, we used Bonferroni corrected *p*-values for multiple comparisons. In logistic regression analysis, all variables such as demographic characteristics, PEL, PA, and school adaptation were used as independent variables, and MHP was used as the outcome variable. The total score of SCL-90 was divided into 4 levels: 0–160 (none), 160–200 (mild), 200–250 (moderate), and >250 (severe). We used ordinal logistic regression to analyze the association between PEL, PA frequency, school adaptation, and specific psychological problems, such as somatization, depression, and hostility. Mean scores on SCL-90 subscales were divided into 4 levels: 1–2 (none), 2–3 (mild), 3–4 (moderate), and 4–5 (severe). Odds ratios and their 95% confidence intervals obtained from the model were reported. A *p*-value <0.05 was considered statistically significant, and all analyses were conducted using SPSS version 22.0 (IBM Corp).

## 3. Results

A total of 9398 high school students (4524, 48.1% boys; 4874, 51.9% girls) were included in the final statistical analysis of this study. The demographic characteristics of the participants were summarized in Table 1. Overall, a total of 7205 (76.7%) students came from urban areas, 2820 (30.0%) students were only children, and 23.7% reported having intimate friends of the opposite sex. In addition, 1786 (19.0%) were from the top classes, and 1452 (15.5%) students reported that their family economic status was poor. The students whose fathers’ and mothers’ educational level was junior high school accounted for 40.0% and 37.5%, respectively.

Respondents’ mean score on the SCL-90 was 137.63 (SD = 453.39). The mean GSI was 1.53 (SD = 0.59). The overall positive rate of mental health problems was 27.0% (Table 2). About 11% reported positive symptoms on at least one of the nine subscales (subscale score ≥ 3). The top three MHP reported were obsessive–compulsive disorder (27.2%), interpersonal sensitivity (20.7%), and depression (18.6%). Somatization, phobic anxiety, and hostility were less common among high school students, with positive rates of 12.4%, 13.1%, and 13.2%, respectively.

In addition, we used the SCL-90 total score as the dependent variable and used multiple logistic regression to analyze the relationship between MHP and various factors. The dependent variable was classified into the same four grades (normal, mild, moderate and severe) as in Table 2, with severe mental health problems as reference. The logistic regression analysis results suggested that there were several important relationships between the variables (Table 3). See Supplementary Materials S2 for further experimental details. Female students thought their probability of having MHP were 1.20 times (OR 1.20, 95% CI 1.09–1.33) than that of male students. Participants without intimate friends of the opposite sex had a 32% (OR 0.68, 95% CI 0.61–0.76) lower chance of developing MHP than those of participants with intimate friends of the opposite sex. Additionally, students from middle-income families were 38% (OR 0.62, 95% CI 0.55–0.70) less likely to have MHP than those from low-income families.

Additionally, with the total score of the SCL-90 subscale as the dependent variable, we used multiple logistic regression to analyze the relationship between each psychological symptom and PEL, PA, and school adaptation. The dependent variables were classified into the same four grades (normal, mild, moderate, and severe) as in Table 2. Furthermore, severe psychological symptoms such as severe somatization and severe obsessive–compulsive disorder were used as references. The results of multiple logistic regression analysis of SCL-90 subscales are presented in Table 4, and more experimental details are in Supplementary Materials S3. The probabilities of having interpersonal sensitivity and anxiety for students whose fathers had a bachelor’s degree were 29% (OR 0.71, 95% CI 0.56–0.91; OR 0.71, 95% CI 0.55–0.93, respectively), lower than those of students whose fathers were in primary education and below. Students’ odds of having obsessive-compulsive disorder, interpersonal sensitivity, and depression were inversely related to their mother’s educational level. Students who were physically active 5–6 times a week had a 60% (OR 0.40, 95% CI 0.31–0.51) lower odds of having depression than students who did not exercise. Compared to students with poor peer relations, students with good peer relations had a 56% (OR 0.44, 95% CI 0.34–0.57) and 52% (OR 0.48, 95% CI 0.37–0.62) lower probability of having psychoticism and somatization, respectively. The probability of having interpersonal sensitivity and obsessive–compulsive disorder for students with good academic behavior were 55% (OR 0.45, 95% CI 0.36–0.55) and 51% (OR 0.49, 95% CI 0.41–0.59) lower than students with poor academic behavior. Participants with high self-management skills reported lower odds of having depression, anxiety, hostility, and psychosis than those with low self-management skills. However, students with high aggressive behaviors thought that their chances of having interpersonal sensitivity were 6.45 times (OR 6.45, 95% CI 1.91–21.75) than students with low aggressive behaviors. The probabilities of having depression for students with high disruptive behaviors were 8.06 times (OR 8.06, 95% CI 2.51–25.81) higher than those with low disruptive behaviors.

## 4. Discussion

The purpose of this study was to examine the associations between PEL, PA, school adaptation, and MHP among Chinese high school students, taking into account demographic factors such as gender, place of residence, siblings, and family economic status. We had several important findings.

First, we found that MHP is common among Chinese high school students. A total of 27.0% of the participants reported positive MHP based on the total SCL-90 score, and the most common symptoms were obsessive–compulsive disorder, interpersonal sensitivity, and depression. From October 2014 to March 2015, Li F et al. selected 17,524 school children and adolescents aged 6–16 years old from five provinces in China for a psychiatric epidemiological survey. The results showed that the prevalence of mental disorders in Chinese children and adolescents was 17.5% [35]. This finding is much lower than the prevalence in this study. On the one hand, high school students are going through a period of rapid physiological and psychological changes that increase their risk of developing mental disorders [36,37]. On the other hand, this study was conducted during the 2020 novel coronavirus epidemic. The COVID-19 pandemic has led to quarantines and educational disruptions for high school students. Studies have found that seclusion, lack of contact with peers, and increased academic stress also contribute to the increased prevalence of MHP in adolescents [38]. Chinese health authorities should strengthen the development and implementation of MHP preventive interventions for high school students.

Second, the results showed that the high probability of self-reported MHP was related to the following factors: female, having intimate friends of the opposite sex, poor family economic status, father’s educational level of elementary school or below, inactive PA, low social competence, and high antisocial behaviors. In this study, we found that MHPs were significantly associated with gender, intimate friends of the opposite sex, family economic status, father’s educational level, PA, and school adaptation, which was in agreement with previous research. Additionally, this study found that there was no statistically significant difference in high school students’ MHP by place of residence and sibling status, which is inconsistent with previous findings. Previous findings indicated that students from rural areas and those with siblings were more likely to suffer from MHP [39,40]. In 2019, the admission rate of middle school entering high school in Shandong Province was 57.41%, and there was a large gap between urban and rural admission rates. Most of the rural students with poor psychological quality and poor learning ability were eliminated by the senior high school entrance examination. Additionally, these discrepancies may be partly attributable to the fact that only 23.3% of the participants in this study were from rural areas. Regarding the association between sibling status and MHP, evidence has shown that older adolescents have significantly increased positive interactions with siblings during COVID-19, so adolescents with siblings may have good mental health during the pandemic [41,42].

Third, further analysis of the SCL-90 subscale revealed that the prevalence of self-reported psychological symptoms of high school students whose parents had a bachelor’s degree was lower than those whose parents were a primary education or below, and the students’ self-reported odds of having obsessive–compulsive disorder, interpersonal sensitivity, and depression were inversely related to their mother’s educational level. Furthermore, in this study, participants’ self-reported MHPs were significantly associated with PA. PA can help reduce the risk of having various psychological symptoms in high school students [13,43]. However, we found that a higher frequency of PA was not always better. Specifically, the frequency of PA 3–4 times per week was most beneficial for somatization and phobic anxiety. For other psychological problems in this study, a frequency of PA 5–6 times per week was optimal. In addition, the findings of the present study revealed that high school students with low learning skills were more prone to have various psychological symptoms, and good peer relations can effectively decrease the odds of psychological symptoms. China’s higher education resources are in short supply, and the competition for college admissions is extremely fierce, which puts forward higher requirements for students’ learning ability. Parents’ expectations for their children’s academic performance also make Chinese high school students face more academic challenges and higher pressure [44,45]. The psychological and behavioral characteristics of adolescents are influenced by their peers, and peer support contributes to mental health [18,46]. Good peer relationships can build strong social networks, such as making more close friends and participating in community activities, which can help high school students cope with challenges and difficulties in study and life and reduce psychological pressure. Last but not least, this study showed that students with high disruptive behaviors had significantly higher rates of psychological symptoms, with the exception of phobic anxiety. Disruptive behaviors have lifetime consequences, such as school absences, poor school achievement, and substance abuse [47]. Therefore, this study suggests that teachers and parents pay more attention to students with disruptive behaviors for early identification and treatment.

We used a large random sample and well-validated questionnaires to preliminarily explore the association between PEL, PA, and school adaptation and MHP among Chinese high school students. This could ensure the reliability and validity of the method and avoid errors that arise from testing a small number of potentially atypical samples. Additionally, we used multiple multidimensional scales that could simultaneously evaluate the impact of a broad range of intervention factors on MHP. The study is significant because the findings could provide a reference for the development of targeted interventions for specific psychological problems of Chinese high school students. This study also has some limitations. First, this study is based on participants’ self-reports, which may be subject to self-report bias. Moreover, the participants were all recruited from Shandong province, so the findings cannot be generalized to the whole high school student population in China. Second, we did not consider other demographic factors, such as parent–child relationships, smoking, alcohol consumption, gaming addiction, and family history of psychotic disorders, which may be associated with high school students’ mental health problems [33,48]. Third, it must be noted that this research is a cross-sectional study, which cannot reflect the development and changes of students’ mental health problems in high school life. Given the above limitations, longitudinal studies with more diverse sample sources and comprehensive designs are needed in the future to better understand the underlying mechanisms of MHP in Chinese high school students.

## 5. Conclusions

The study found that the proportion of MHP among high school students in Shandong Province is relatively high. We recommended strengthening the prevention, early detection, and treatment of MHP in high school students, especially those who are female, have intimate friends of the opposite sex, have poor family economic conditions, and have low educational attainment of parents. There are significant differences in the effects of PEL, PA, and school adaptation on the MHP of high school students. Fathers with a master’s degree or above should actively participate in their children’s mental health education. More attention should be given to students with antisocial behaviors, especially disruptive behaviors. We suggest that schools and society foster an environment conducive to expanding social networks and strengthening physical exercise and formulate differentiated mental health prevention and intervention strategies for different individuals.

## Figures and Tables

**Table 1:** Participants’ demographic characteristics and results of self-reported mental health problems, N = 9398.

Demographic Characteristics	Number of Participants, *n* (%)	SCL-90 Score (Mean ± SD)	t/F	*p*
**Gender**			t = −5.19	<0.001
Male	4524 (48.1)	134.67 ± 53.73		
Female	4874 (51.9)	140.38 ± 52.93		
**Place of residence**			t = −2.44	0.02
Urban	7205 (76.7)	136.89 ± 54.03		
Rural	2193 (23.3)	140.07 ± 51.15		
**Single child household**			t = −3.09	0.002
Yes	2820 (30.0)	135.03 ± 54.72		
No	6578 (70.0)	138.74 ± 52.77		
**Intimate friends of the opposite sex ^a^**			t = 7.95	<0.001
Have	2230 (23.7)	147.06 ± 57.66		
No	6441 (68.5)	136.07 ± 51.99		
**In the top class**			F = 6.98	0.008
No	7612 (81.0)	136.93 ± 52.76		
Yes	1786 (19.0)	140.63 ± 55.91		
**Family economic status**			F = 108.22	<0.001
Poor	1452 (15.5)	156.09 ± 65.58		
Middle	7219 (76.8)	133.78 ± 49.12		
Good	727 (7.7)	139.01 ± 58.98		
**Father’s educational level**			F = 49.73	<0.001
Elementary school and below	1050 (11.2)	156.07 ± 66.94		
Junior high school	3760 (40.0)	138.59 ± 51.08		
High school	2736 (29.1)	135.50 ± 50.94		
University	1675 (17.8)	127.20 ± 47.90		
Postgraduate and above	177 (1.9)	139.42 ± 66.60		
**Mother’s educational level**			F = 39.02	<0.001
Elementary school and below	2020 (21.5)	148.73 ± 58.66		
Junior high school	3523 (37.5)	137.58 ± 51.54		
High school	2368 (25.2)	134.69 ± 51.87		
University	1322 (14.1)	126.37 ± 47.80		
Postgraduate and above	165 (1.8)	135.29 ± 63.17		
**PA**			t = 10.16	<0.001
Inactive	5107 (54.3)	142.69 ± 55.11		
Active	4291 (45.7)	131.60 ± 50.61		
**SSBS-Social Competence**			F = 411.47	<0.001
Low	1311 (13.9)	165.81 ± 71.75		
Middle	3875 (41.2)	144.34 ± 51.82		
High	4212 (44.8)	122.68 ± 42.14		
**SSBS-Antisocial Behavior**			F = 133.03	<0.001
Low	9211 (98.0)	136.41 ± 51.66		
Middle	158 (1.7)	190.84 ± 78.08		
High	29 (0.3)	235.28 ± 135.06		

^a^ The total number of participants in this category does not equal 9398 due to missing data.

**Table 2:** Prevalence of different levels of mental health problems, *n* (%).

Subscale	Mean ± SD	Normal	Mild	Moderate	Severe
SCL-90 Score	137.63 (53.39)	6863 (73.0)	1465 (15.6)	636 (6.8)	434 (4.6)
GSI Score	1.53 (0.59)	5803 (61.7)	-	-	-
SOM *	1.43 (0.58)	8230 (87.6)	963 (10.2)	161 (1.7)	44 (0.5)
O-C *	1.75 (0.69)	6843 (72.8)	2078 (22.1)	397 (4.2)	80 (0.9)
I-S *	1.61 (0.69)	7448 (79.3)	1545 (16.4)	325 (3.5)	80 (0.9)
DEP *	1.56 (0.68)	7648 (81.4)	1339 (14.2)	321 (3.4)	90 (1.0)
ANX *	1.53 (0.66)	7869 (83.7)	1196 (12.7)	254 (2.7)	79 (0.8)
HOS *	1.47 (0.64)	8161 (86.8)	952 (10.1)	209 (2.2)	76 (0.8)
PHOB *	1.44 (0.63)	8163 (86.9)	964 (10.3)	218 (2.3)	53 (0.6)
PAR *	1.48 (0.63)	8088 (86.1)	1055 (11.2)	204 (2.2)	51 (0.5)
PSY *	1.46 (0.59)	8135 (86.6)	1034 (11.0)	187 (2.0)	42 (0.4)

* SOM, somatization; O-C, obsessive–compulsive; I-S, interpersonal sensitivity; DEP, depression; ANX, anxiety; HOS, hostility; PHOB, phobic anxiety; PAR, paranoid ideation; PSY, psychoticism.

**Table 3:** Results of logistic regression analysis of factors that were associated with mental health problems.

Categories	B	S.E	OR (95% CI)	*p*-Value ^b^
**Gender**				
Male	0 ^a^		1.0 (Referent)	N/A ^c^
Female	0.18	0.05	1.20 (1.09, 1.33)	<0.001
**Place of residence**				
Urban	0 ^a^			N/A ^c^
Rural	−0.03	0.06	0.97 (0.87, 1.10)	0.652
**Single child household**				
Yes	0 ^a^		1.0 (Referent)	N/A ^c^
No	0.11	0.06	1.12 (1.00,1.25)	0.062
**Intimate friends of the opposite sex**				
Have	0 ^a^	0.03	1.0 (Referent)	N/A ^c^
No	−0.38	0.05	0.68 (0.61, 0.76)	<0.001
**In the top class**				
No	0 ^a^		1.0 (Referent)	N/A ^c^
Yes	0.09	0.06	1.09 (0.97, 1.23)	0.166
**Family economic status**				
Poor	0 ^a^		1.0 (Referent)	N/A ^c^
Middle	−0.48	0.07	0.62 (0.55, 0.70)	<0.001
Good	−0.23	0.11	0.80 (0.65, 0.99)	0.038
**Father’s educational level**				
Elementary school and below	0 ^a^		1.0 (Referent)	N/A ^c^
Junior high school	−0.14	0.08	0.87 (0.74, 1.02)	0.077
High school	−0.13	0.09	0.88 (0.74, 1.05)	0.148
University	−0.25	0.12	0.78 (0.62, 0.98)	0.033
Graduate and above	−0.12	0.28	0.88 (0.50, 1.53)	0.658
**Mother’s educational level**				
Elementary school and below	0 ^a^		1.0 (Referent)	N/A ^c^
Junior high school	−0.08	0.07	0.92 (0.81, 1.05)	0.222
High school	−0.12	0.08	0.88 (0.75, 1.04)	0.131
University	−0.20	0.12	0.82 (0.65, 1.03)	0.084
Graduate and above	−0.28	0.28	0.75 (0.43, 1.32)	0.319
**PA**				
Inactive	0 ^a^		1.0 (Referent)	N/A ^c^
Active	−0.26	0.05	0.77 (0.70, 0.85)	<0.001
**SSBS-Social Competence**				
Low	0 ^a^		1.0 (Referent)	N/A ^c^
Middle	−0.56	0.07	0.57 (0.50, 0.65)	<0.001
High	−1.47	0.07	0.23 (0.20, 0.27)	<0.001
**SSBS-Antisocial Behavior**				
Low	0 ^a^		1.0 (Referent)	N/A ^c^
Middle	1.31	0.16	3.71 (2.72, 5.05)	<0.001
High	1.94	0.37	6.98 (3.41, 14.32)	<0.001

^a^ The total number of participants in this category does not equal 9398 due to missing data. ^b^ Bonferroni *p*-values are used to correct for multiple comparisons. Statistical significance was set to a level of *p* < 0.05. ^c^ N/A: not appliable.

**Table 4:** Results of multiple logistic regression analysis on SCL-90 subscales.

Categories	SOM	O-C	I-S	DEP	ANX	HOS	PHOB	PAR	PSY
**Father’s educational level**									
Elementary school and below	1.0 (Referent)	1.0 (Referent)	1.0 (Referent)	1.0 (Referent)	1.0 (Referent)	1.0 (Referent)	1.0 (Referent)	1.0 (Referent)	1.0 (Referent)
Junior high school	0.79 (0.65, 0.97) *	0.82 (0.70, 0.96) *	0.77 (0.65, 0.90) **	0.76 (0.64, 0.90) **	0.77 (0.64, 0.93) **	0.78 (0.64, 0.95) *	0.80 (0.66, 0.97) *	0.76 (0.63, 0.92) **	0.70 (0.58, 0.86) ***
High school	0.83 (0.66, 1.04)	0.78 (0.66, 0.93) **	0.76 (0.63, 0.92) **	0.79 (0.65, 0.95) *	0.81 (0.66, 0.99) *	0.77 (0.62, 0.97) *	0.76 (0.61, 0.95) *	0.75 (0.60, 0.93) **	0.70 (0.56, 0.87) **
University	0.77 (0.57, 1.04)	0.75 (0.60, 0.93) **	0.71 (0.56, 0.91) **	0.76 (0.59, 0.98) *	0.71 (0.55, 0.93) *	0.85 (0.64, 1.13)	0.75 (0.56, 0.99) *	0.82 (0.62, 1.09)	0.74 (0.55, 0.98) *
Graduate and above	1.08 (0.59, 1.98)	0.71(0.42, 1.21)	0.93 (0.54, 1.59)	0.77 (0.43, 1.38)	0.83 (0.46, 1.50)	0.68 (0.36, 1.28)	0.75 (0.39, 1.43)	1.16 (0.64, 2.10)	0.89 (0.48, 1.67)
**Mother’s educational level**									
Elementary school and below	1.0 (Referent)	1.0 (Referent)	1.0 (Referent)	1.0 (Referent)	1.0 (Referent)	1.0 (Referent)	1.0 (Referent)	1.0 (Referent)	1.0 (Referent)
Junior high school	0.90 (0.76, 1.07)	0.90 (0.79, 1.02)	0.87 (0.75, 1.00) *	0.83 (0.72, 0.96) *	0.85 (0.73, 0.99) *	0.86 (0.72, 1.01)	0.91 (0.77, 1.08)	0.86 (0.73, 1.01)	0.90 (0.76, 1.07)
High school	0.83 (0.68, 1.02)	0.70 (0.60, 0.82) ***	0.73 (0.62, 0.87) ***	0.63 (0.53, 0.75) ***	0.78 (0.65, 0.94) **	0.74 (0.60, 0.91) **	0.79 (0.65, 0.97) *	0.81 (0.67, 0.99) *	0.83 (0.68, 1.02)
University	0.61 (0.45, 0.83) **	0.59 (0.48, 0.73) ***	0.59 (0.46, 0.75) ***	0.59 (0.46, 0.76) ***	0.58 (0.45, 0.77) ***	0.64 (0.48, 0.86) **	0.66 (0.49, 0.88) **	0.64 (0.49, 0.86) **	0.60 (0.45, 0.80) **
Graduate and above	0.77 (0.41, 1.43)	0.41 (0.24, 0.72) **	0.47 (0.26, 0.84) *	0.44 (0.24, 0.81) **	0.54 (0.28, 1.00)	0.73 (0.39, 1.39)	0.63 (0.33, 1.23)	0.49 (0.26, 0.93) *	0.49 (0.25, 0.95) *
**PA**									
0 times pw	1.0 (Referent)	1.0 (Referent)	1.0 (Referent)	1.0 (Referent)	1.0 (Referent)	1.0 (Referent)	1.0 (Referent)	1.0 (Referent)	1.0 (Referent)
1–2 times pw	0.59 (0.51, 0.68) ***	0.60 (0.54, 0.67) ***	0.55 (0.49, 0.62) ***	0.52 (0.46, 0.59) ***	0.58 (0.51, 0.66) ***	0.62 (0.53, 0.71) ***	0.59 (0.52, 0.69) ***	0.53 (0.46, 0.61) ***	0.51 (0.44, 0.59) ***
3–4 times pw	0.54 (0.44, 0.66) ***	0.52 (0.45, 0.61) ***	0.46 (0.39, 0.55) ***	0.44 (0.37, 0.52) ***	0.55 (0.46, 0.65) ***	0.60 (0.50, 0.73) ***	0.51 (0.41, 0.62) ***	0.54 (0.45, 0.66) ***	0.51 (0.42, 0.62) ***
5–6 times pw	0.57 (0.43, 0.74) ***	0.51 (0.42, 0.63) ***	0.45 (0.36, 0.56) ***	0.40 (0.31, 0.51) ***	0.49 (0.38, 0.63) ***	0.54 (0.41, 0.71) ***	0.54 (0.42, 0.71) ***	0.47 (0.36, 0.62) ***	0.45 (0.34, 0.59) ***
≥7 times pw	0.77 (0.56, 1.04)	0.75 (0.59, 0.95) *	0.70 (0.55, 0.91) **	0.70 (0.54, 0.91) **	0.67 (0.50, 0.89) **	0.72 (0.53, 0.99) *	0.70 (0.51, 0.95) *	0.71 (0.53, 0.96) *	0.62 (0.45, 0.85) **
**Social Competence**									
**Peer relations**									
Low	1.0 (Referent)	1.0 (Referent)	1.0 (Referent)	1.0 (Referent)	1.0 (Referent)	1.0 (Referent)	1.0 (Referent)	1.0 (Referent)	1.0 (Referent)
Middle	0.78 (0.64, 0.96) *	1.01 (0.86, 1.19)	0.87 (0.73, 1.03)	0.92 (0.77, 1.10)	0.88 (0.73, 1.05)	0.77 (0.63, 0.93) **	0.88 (0.72, 1.07)	0.72 (0.60, 0.88) **	0.73 (0.60, 0.89) **
High	0.48 (0.37, 0.62) ***	0.67 (0.55, 0.82) ***	0.58 (0.47, 0.72) ***	0.65 (0.52, 0.81) ***	0.58 (0.46, 0.74) ***	0.57 (0.44, 0.73) ***	0.61 (0.47, 0.79) ***	0.49 (0.38, 0.63) ***	0.44(0.34, 0.57) ***
**Self management/Compliance**									
Low	1.0 (Referent)	1.0 (Referent)	1.0 (Referent)	1.0 (Referent)	1.0 (Referent)	1.0 (Referent)	1.0 (Referent)	1.0 (Referent)	1.0 (Referent)
Middle	0.88 (0.71, 1.09)	1.15 (0.96, 1.38)	1.02 (0.85, 1.23)	0.80 (0.66, 0.97) *	0.82 (0.68, 1.00)	0.84 (0.68, 1.03)	0.92 (0.74, 1.13)	0.86 (0.70, 1.05)	0.80 (0.65, 0.98) *
High	0.85 (0.65, 1.12)	1.11 (0.89, 1.38)	0.90 (0.71, 1.13)	0.64 (0.50, 0.81) ***	0.69 (0.54, 0.88) **	0.58 (0.44, 0.76) ***	0.80 (0.61, 1.05)	0.69 (0.53, 0.90) **	0.66 (0.50, 0.86) **
**Academic behavior**									
Low	1.0 (Referent)	1.0 (Referent)	1.0 (Referent)	1.0 (Referent)	1.0 (Referent)	1.0 (Referent)	1.0 (Referent)	1.0 (Referent)	1.0 (Referent)
Middle	0.78 (0.66, 0.94) **	0.74 (0.65, 0.85) ***	0.70 (0.61, 0.81) ***	0.74 (0.63, 0.86) ***	0.78 (0.67, 0.91) **	0.72 (0.61, 0.86) ***	0.71 (0.60, 0.84) ***	0.76 (0.64, 0.89) **	0.78 (0.66, 0.93) **
High	0.67 (0.52, 0.85) **	0.49 (0.41, 0.59) ***	0.45 (0.36, 0.55) ***	0.52 (0.42, 0.64) ***	0.56 (0.44, 0.69) ***	0.55 (0.43, 0.70) ***	0.52 (0.41, 0.66) ***	0.58 (0.46, 0.74) ***	0.63 (0.49, 0.80) ***
**Antisocial Behavior**									
**Hostile/Irritable**									
Low	1.0 (Referent)	1.0 (Referent)	1.0 (Referent)	1.0 (Referent)	1.0 (Referent)	1.0 (Referent)	1.0 (Referent)	1.0 (Referent)	1.0 (Referent)
Middle	2.46 (1.79,3.37) ***	1.47 (1.09, 1.98) *	1.66 (1.22, 2.26) **	2.04 (1.50, 2.76) ***	2.25 (1.66, 3.06) ***	2.04 (1.49, 2.81) ***	2.44 (1.78, 3.35) ***	2.66 (1.96, 3.62) ***	2.20 (1.61, 3.02) ***
High	0.97(0.35,2.68)	0.39 (0.14, 1.10)	0.42 (0.14, 1.26)	0.68 (0.24, 1.91)	0.70 (0.24, 2.03)	0.82 (0.28, 2.36)	0.85 (0.29, 2.44)	1.05 (0.38, 2.90)	1.73 (0.67, 4.45)
**Antisocial/Aggressive**									
Low	1.0 (Referent)	1.0 (Referent)	1.0 (Referent)	1.0 (Referent)	1.0 (Referent)	1.0 (Referent)	1.0 (Referent)	1.0 (Referent)	1.0 (Referent)
Middle	1.51 (1.05, 2.17) *	1.15(0.82, 1.63)	1.14(0.80, 1.61)	1.11 (0.78, 1.58)	1.27 (0.89, 1.81)	1.49 (1.04, 2.14) *	1.24 (0.85, 1.80)	1.15 (0.80, 1.66)	1.31 (0.91, 1.87)
High	2.64 (0.78, 8.92)	3.04 (0.90, 10.20)	6.45(1.91, 21.75) **	1.95 (0.57, 6.63)	3.08 (0.91, 10.47)	5.37 (1.60, 18.01) **	4.31 (1.28, 14.55) *	2.91 (0.87, 9.67)	1.60 (0.47, 5.40)
**Defiant/Disruptive**									
Low	1.0 (Referent)	1.0 (Referent)	1.0 (Referent)	1.0 (Referent)	1.0 (Referent)	1.0 (Referent)	1.0 (Referent)	1.0 (Referent)	1.0 (Referent)
Middle	2.09 (1.48, 2.94) ***	1.51 (1.09, 2.09) *	1.60 (1.15, 2.22) **	1.99 (1.44, 2.77) ***	1.63 (1.17, 2.28) **	1.92 (1.37, 2.69) ***	1.95 (1.38, 2.76) ***	1.97 (1.41, 2.76) ***	2.18 (1.56, 3.05) ***
High	3.74 (1.18, 11.9) *	7.16 (2.26, 22.71) **	3.75 (1.21, 11.67) *	8.06 (2.51, 25.81) **	6.25 (1.97, 19.82) **	4.53 (1.46, 14.06) *	3.18 (1.00, 10.07)	6.06 (1.94, 18.98) **	4.15 (1.30, 13.29) *

SOM, somatization; O-C, obsessive–compulsive; I-S, interpersonal sensitivity; DEP, depression; ANX, anxiety; HOS, hostility; PHOB, phobic anxiety; PAR, paranoid ideation; PSY, psychoticism. * *p* < 0.05, ** *p* < 0.01, *** *p* < 0.001.

## Data Availability

The data presented in this study are openly available in the Population Health Data Archive [27].

## Supplementary Materials

The following supporting information can be downloaded at: https://www.mdpi.com/article/10.3390/ijerph19148478/s1.
