# Supplementary material for: Mental Health Problems and Associated Factors among High School Students in Shandong Province of China: A Cross-Sectional Study
Source: Int J Environ Res Public Health. 2022 Jul 11;19(14):8478. doi: 10.3390/ijerph19148478 (PMC9320481; doi:10.3390/ijerph19148478)
Supplement: Supplementary file 1 [file ijerph-19-08478-s001.zip › ijerph-1750265-supplementary.pdf]

Mental Health Problems and Associated Factors among High School Students in Shandong Province of China: A Cross-Sectional Study

A

Follow-up analysis for the reliability analysis of PAQ-A, SSBS-2 and SCL-90 scales

Table S1. The reliability and validity analysis of PAQ-A.

	α	ω	AVE	CR	X^2/df	GFI	RMSEA	CFI	NFI	TLI	SRMR
PAQ-A	0.817	0.872	0.481	0.819	55.779	0.988	0.076	0.982	0.982	0.964	0.022

α, Cronbach's alpha; ω, McDonald's omega; AVE, Average Variance Extracted; CR, Composite Reliability; GFI, Goodness Fit Index; RMSEA, Root Mean Square Error of Approximation; CFI, Comparative Fit Index; NNFI, Non-Normed Fit Index; TLI, Tucker–Lewis Index; SRMR, Standardized Root Mean Square Residual.

Table S2. The reliability and validity analysis of SSBS-2.

Subscale	α	ω	AVE	CR	X^2/df	GFI	RMSEA	CFI	NFI	TLI	SRMR
Peer relations	0.935	0.945	0.518	0.937	123.109	0.862	0.114	0.885	0.884	0.864	0.052
Self management/ Compliance	0.912	0.928	0.517	0.914	107.364	0.919	0.106	0.926	0.925	0.904	0.043
Academic behavior	0.912	0.929	0.567	0.913	59.464	0.970	0.079	0.972	0.971	0.961	0.026
Hostile/Irritable	0.917	0.934	0.504	0.924	85.043	0.907	0.095	0.925	0.924	0.909	0.046
Anti-social/ Aggressive	0.914	0.931	0.527	0.917	104.877	0.915	0.105	0.929	0.928	0.909	0.045
Defiant/Disruptive	0.902	0.924	0.522	0.907	206.316	0.855	0.148	0.880	0.879	0.839	0.063

α, Cronbach's alpha; ω, McDonald's omega; AVE, Average Variance Extracted; CR, Composite Reliability; GFI, Goodness Fit Index; RMSEA, Root Mean Square Error of Approximation; CFI, Comparative Fit Index; NNFI, Non-Normed Fit Index; TLI, Tucker–Lewis Index; SRMR, Standardized Root Mean Square Residual.

Table S3. The reliability and validity analysis of SCL-90.

Subscale	α	ω	AVE	CR	X^2/df	GFI	RMSEA	CFI	NNFI	TLI	SRMR
Somatization	0.922	0.935	0.505	0.924	52.261	0.948	0.074	0.953	0.952	0.943	0.034
Obsessive-compulsive	0.897	0.915	0.470	0.898	68.532	0.948	0.085	0.943	0.942	0.927	0.039
Interpersonal sensitivity	0.898	0.919	0.507	0.901	48.597	0.969	0.071	0.969	0.968	0.958	0.026
Depression	0.935	0.945	0.538	0.937	58.914	0.932	0.079	0.950	0.949	0.940	0.031
Anxiety	0.920	0.935	0.550	0.924	45.843	0.965	0.069	0.971	0.970	0.963	0.026
Postility	0.873	0.907	0.544	0.877	145.855	0.956	0.124	0.950	0.950	0.917	0.037
Phobic anxiety	0.870	0.901	0.495	0.872	53.913	0.977	0.075	0.972	0.972	0.958	0.029
Paranoid ideation	0.850	0.890	0.492	0.852	23.092	0.993	0.048	0.990	0.990	0.984	0.017
Psychoticism	0.896	0.914	0.466	0.896	35.537	0.972	0.061	0.970	0.969	0.961	0.029
Additional items	0.848	0.885	0.488	0.826	155.068	0.966	0.128	0.952	0.951	0.903	0.038

α, Cronbach's alpha; ω, McDonald's omega; AVE, Average Variance Extracted; CR, Composite Reliability; GFI, Goodness Fit Index; RMSEA, Root Mean Square Error of Approximation; CFI, Comparative Fit Index; NNFI, Non-Normed Fit Index; TLI, Tucker–Lewis Index; SRMR, Standardized Root Mean Square Residual.

B

Follow-up analysis for Table 3 (Results of logistic regression analysis of factors that were associated with mental health problems) in the main text.

Table S4. Case Processing Summary.

	N	Percent
Included	8671	92.3%
Excluded	727	7.7%
Total	9398	100.0%

Table S5. Goodness of Fit.

	Value	df	Value/df
Deviance	5781.938	7330	0.789
Scaled Deviance	5781.938	7330	
Pearson Chi-Square	7846.977	7330	1.071
Scaled Pearson Chi-Square	7846.977	7330	
Log Likelihood	-3974.929		
Akaike's Information Criterion (AIC)	7995.858		
Finite Sample Corrected AIC (AICC)	7995.986		
Bayesian Information Criterion (BIC)	8158.416		
Consistent AIC (CAIC)	8181.416		

Table S6. Omnibus Test.

Likelihood Ratio Chi-Square	df	Sig.
889.340	20	0.000

Table S7. SCL-90 level * Predicted Response Category Crosstabulation.

			Predicted Response Category			Total
			Normal	Mild	Severe	
SCL-90 level	Normal	Count	6239	5	12	6256
		% within SCL-90 level	99.7%	0.1%	0.2%	100.0%
	Mild	Count	1392	5	13	1410
		% within SCL-90 level	98.7%	0.4%	0.9%	100.0%
	Moderate	Count	580	7	5	592
		% within SCL-90 level	98.0%	1.2%	0.8%	100.0%
	Severe	Count	389	4	20	413
		% within SCL-90 level	94.2%	1.0%	4.8%	100.0%
Total		Count	8600	21	50	8671
		% within SCL-90 level	99.2%	0.2	0.6%	100.0%

Table S8. Parameter Estimates.

Parameter		B	Std. Error	95% Wald Confidence Interval		Hypothesis Test			Exp(B)	95% Wald Confidence Interval for Exp(B)	
				Lower	Upper	Wald Chi-Square	df	Sig.		Lower	Upper
Threshold	[SCL90 level = Normal]	-0.649	0.1107	-0.866	-0.432	34.402	1	0.000	0.522	0.421	0.649
	[SCL90 level = Mild]	0.523	0.1111	0.306	0.741	22.188	1	0.000	1.688	1.357	2.098
	[SCL90 level = Moderate]	1.544	0.1162	1.316	1.772	176.416	1	0.000	4.683	3.729	5.881
**Gender**											
Female		0.184	0.0507	0.084	0.283	13.083	1	0.000	1.201	1.088	1.327
Male		0[a]	.	.	.	.	.	.	1	.	.
**Place of residence**											
Rural		-0.027	0.0601	-0.145	0.091	0.203	1	0.652	0.973	0.865	1.095
Urban		0[a]	.	.	.	.	.	.	1	.	.
**Single child household**											
No		0.110	0.0590	-0.005	0.226	3.489	1	0.062	1.116	0.995	1.253
Yes		0[a]	.	.	.	.	.	.	1	.	.
**Intimate friends of the opposite sex**											
No		-0.382	0.0542	-0.488	-0.276	49.713	1	0.000	0.682	0.614	0.759
Have		0[a]	.	.	.	.	.	.	1	.	.
**Family economic status**											
Good		-0.226	0.1085	-0.438	-0.013	4.322	1	0.038	0.798	0.645	0.987
Middle		-0.481	0.0644	-0.607	-0.355	55.818	1	0.000	0.618	0.545	0.701
Poor		0[a]	.	.	.	.	.	.	1	.	.
**In the top class**											
Yes		0.085	0.0613	-0.035	0.205	1.920	1	0.166	1.089	0.965	1.228
No		0[a]	.	.	.	.	.	.	1	.	.
**Father's educational level**											
Graduate and above		-0.124	0.2805	-0.674	0.426	0.196	1	0.658	0.883	0.510	1.531
University		-0.248	0.1164	-0.476	-0.020	4.550	1	0.033	0.780	0.621	0.980

High school	-0.131	0.0903	-0.308	0.046	2.095	1	0.148	0.877	0.735	1.047
Junior high school	-0.144	0.0811	-0.303	0.015	3.135	1	0.077	0.866	0.739	1.015
Elementary school and below	0[a]	.	.	.	.	.	.	1	.	.
**Mother's educational level**										
Graduate and above	-0.284	0.2847	-0.842	0.274	0.992	1	0.319	0.753	0.431	1.316
University	-0.200	0.1158	-0.427	0.027	2.991	1	0.084	0.818	0.652	1.027
High school	-0.123	0.0816	-0.283	0.037	2.277	1	0.131	0.884	0.753	1.038
Junior high school	-0.082	0.0671	-0.214	0.050	1.493	1	0.222	0.921	0.808	1.051
Elementary school and below	0[a]	.	.	.	.	.	.	1	.	.
**PA**										
Active	-0.263	0.0511	-0.363	-0.163	26.594	1	0.000	0.768	0.695	0.849
Inactive	0[a]	.	.	.	.	.	.	1	.	.
**SSBS-Social Competence**										
High	-1.465	0.0729	-1.608	-1.322	404.125	1	0.000	0.231	0.200	0.267
Middle	-0.564	0.0674	-0.696	-0.432	70.014	1	0.000	0.569	0.499	0.650
Low	0[a]	.	.	.	.	.	.	1	.	.
**SSBS-Antisocial Behavior**										
High	1.944	0.3664	1.225	2.662	28.136	1	0.000	6.984	3.406	14.321
Middle	1.310	0.1577	1.001	1.619	69.029	1	0.000	3.707	2.721	5.049
Low	0[a]	.	.	.	.	.	.	1	.	.
(Scale)	1[b]									

a. Set to zero because this parameter is redundant.
b. Fixed at the displayed value.

C

Follow-up analysis for Table 4 (Results of multiple logistic regression analysis on SCL-90 subscales) in the main text.

Table S9. Case Processing Summary.

	N	Percent
Included	9398	100.0%
Excluded	0	0.0%
Total	9398	100.0%

C.1 Somatization

Table S10. Goodness of Fit.

	Value	df	Value/df
Deviance	2359.081	4149	0.569
Scaled Deviance	2359.081	4149	
Pearson Chi-Square	4623.940	4149	1.114
Scaled Pearson Chi-Square	4623.940	4149	
Log Likelihoodb	-1684.322		
Akaike's Information Criterion (AIC)	3422.644		
Finite Sample Corrected AIC (AICC)	3422.805		
Bayesian Information Criterion (BIC)	3615.646		
Consistent AIC (CAIC)	3642.646		

Table S11. Omnibus Test.

Likelihood Ratio Chi-Square	df	Sig.
574.987	24	0.000

Table S12. Parameter Estimates.

Parameter		B	Std. Error	95% Wald Confidence Interval		Hypothesis Test			Exp(B)	95% Wald Confidence Interval for Exp(B)	
				Lower	Upper	Wald Chi-Square	df	Sig.		Lower	Upper
Threshold	[Somatization level = Normal]	0.567	0.1136	0.344	0.790	24.924	1	0.000	1.763	1.411	2.203
	[Somatization level = Mild]	2.509	0.1286	2.257	2.761	380.965	1	0.000	12.297	9.558	15.822
	[Somatization level = Moderate]	4.103	0.1849	3.740	4.465	492.457	1	0.000	60.513	42.119	86.941
**Father's educational level**											
Graduate and above		0.078	0.3092	-0.528	0.684	0.063	1	0.802	1.081	0.590	1.981
University		-0.261	0.1508	-0.557	0.035	2.995	1	0.084	0.770	0.573	1.035
High school		-0.190	0.1150	-0.416	0.035	2.741	1	0.098	0.827	0.660	1.036
Junior high school		-0.233	0.1040	-0.437	-0.029	5.004	1	0.025	0.792	0.646	0.972
Elementary school and below		0[a]	.	.	.	.	.	.	1	.	.
**Mother's educational level**											
Graduate and above		-0.266	0.3193	-0.892	.360	0.695	1	0.404	0.766	0.410	1.433
University		-0.495	0.1549	-0.799	-0.191	10.209	1	0.001	0.610	0.450	0.826
High school		-0.187	0.1050	-0.393	0.019	3.170	1	0.075	0.830	0.675	1.019
Junior high school		-0.102	0.0882	-0.275	0.071	1.343	1	0.247	0.903	0.759	1.073
Elementary school and below		0[a]	.	.	.	.	.	.	1	.	.
**PA**											
≥7 time pw		-0.267	0.1568	-0.575	0.040	2.903	1	0.088	0.766	0.563	1.041
5-6 time pw		-0.569	0.1399	-0.844	-0.295	16.552	1	0.000	0.566	0.430	0.744
3-4 time pw		-0.617	0.1027	-0.819	-0.416	36.122	1	0.000	0.539	0.441	0.660
1-2 time pw		-0.528	0.0759	-0.677	-0.379	48.457	1	0.000	0.590	0.508	0.684
0 time pw		0[a]	.	.	.	.	.	.	1	.	.
**Social Competence**											
**Peer relations**											
High		-0.744	0.1348	-1.008	-0.479	30.420	1	0.000	0.475	0.365	0.619

Middle	-0.245	.1035	-0.448	-0.042	5.595	1	0.018	0.783	0.639	0.959
Low	0[a]	.	.	.	.	.	.	1	.	.
**Self management/Compliance**										
High	-0.162	0.1413	-0.439	0.115	1.310	1	0.252	0.851	0.645	1.122
Middle	-0.126	0.1099	-0.341	0.089	1.317	1	0.251	0.882	0.711	1.093
Low	0[a]	.	.	.	.	.	.	1	.	.
**Academic behavior**										
High	-0.407	0.1263	-0.654	-0.159	10.352	1	0.001	0.666	0.520	0.853
Middle	-0.245	0.0906	-0.422	-0.067	7.285	1	0.007	0.783	0.656	0.935
Low	0[a]	.	.	.	.	.	.	1	.	.
**Antisocial Behavior**										
**Hostile/Irritable**										
High	-0.034	0.5198	-1.053	0.985	0.004	1	0.948	0.967	0.349	2.678
Middle	0.898	0.1613	0.582	1.214	30.999	1	0.000	2.455	1.790	3.369
Low	0[a]	.	.	.	.	.	.	1	.	.
**Anti-social/Aggressive**										
High	0.970	0.6215	-0.248	2.188	2.437	1	0.118	2.639	0.780	8.921
Middle	0.412	0.1855	0.048	0.775	4.929	1	0.026	1.509	1.049	2.171
Low	0[a]	.	.	.	.	.	.	1	.	.
**Defiant/Disruptive**										
High	1.319	0.5910	0.161	2.477	4.983	1	0.026	3.740	1.175	11.911
Middle	0.737	0.1745	0.395	1.079	17.839	1	0.000	2.090	1.484	2.942
Low	0[a]	.	.	.	.	.	.	1	.	.
(Scale)	1[b]									

a. Set to zero because this parameter is redundant.
b. Fixed at the displayed value.

C.2 Obsessive-compulsive

Table S13. Goodness of Fit.

	**Value**	**df**	**Value/df**
Deviance	3072.809	4149	0.741
Scaled Deviance	3072.809	4149	
Pearson Chi-Square	5312.606	4149	1.280
Scaled Pearson Chi-Square	5312.606	4149	
Log Likelihoodb	-2341.790		
Akaike's Information Criterion (AIC)	4737.580		
Finite Sample Corrected AIC (AICC)	4737.741		
Bayesian Information Criterion (BIC)	4930.582		
Consistent AIC (CAIC)	4957.582		

Table S14. Omnibus Test.

**Likelihood Ratio Chi-Square**	**df**	**Sig.**
732.371	24	0.000

Table S15. Parameter Estimates.

Parameter	B	Std. Error	95% Wald Confidence Interval		Hypothesis Test			Exp(B)	95% Wald Confidence Interval for Exp(B)	
			Lower	Upper	Wald Chi-Square	df	Sig.		Lower	Upper
Threshold [Obsessive-compulsive level = Normal]	-0.249	0.0947	-0.435	-0.063	6.917	1	0.009	0.780	0.647	0.939
[Obsessive-compulsive level = Mild]	1.794	0.1009	1.596	1.992	316.106	1	0.000	6.013	4.934	7.328
[Obsessive-compulsive level = Moderate]	3.672	0.1436	3.391	3.953	654.189	1	0.000	39.335	29.687	52.117
**Father's educational level**										
Graduate and above	-0.336	.2683	-0.862	0.190	1.571	1	0.210	0.714	0.422	1.209
University	-0.293	.1121	-0.513	-0.074	6.852	1	0.009	0.746	0.599	0.929
High school	-0.249	0.0880	-0.421	-0.076	7.989	1	0.005	0.780	0.656	0.927
Junior high school	-0.197	0.0790	-0.352	-0.043	6.248	1	0.012	0.821	0.703	0.958
Elementary school and below	0[a]	.	.	.	.	.	.	1	.	.
**Mother's educational level**										
Graduate and above	-0.882	.2851	-1.441	-0.323	9.575	1	0.002	0.414	0.237	0.724
University	-0.528	.1106	-0.745	-0.311	22.771	1	0.000	0.590	0.475	0.733
High school	-0.356	0.0788	-0.510	-0.202	20.413	1	0.000	0.701	0.600	0.817
Junior high school	-0.109	0.0645	-0.235	0.018	2.827	1	0.093	0.897	0.791	1.018
Elementary school and below	0[a]	.	.	.	.	.	.	1	.	.
**PA**										
≥7 time pw	-0.287	.1189	-0.520	-0.054	5.811	1	0.016	0.751	0.595	0.948
5-6 time pw	-0.673	.1037	-0.876	-0.470	42.159	1	0.000	0.510	0.416	0.625
3-4 time pw	-0.649	0.0751	-0.796	-0.502	74.633	1	0.000	0.522	0.451	0.605
1-2 time pw	-0.509	0.0568	-0.620	-0.397	80.224	1	0.000	0.601	0.538	0.672
0 time pw	0[a]	.	.	.	.	.	.	1	.	.
**Social Competence**										
**Peer relations**										
High	-0.403	.1027	-0.604	-0.202	15.404	1	0.000	0.668	0.546	0.817

Middle	0.011	0.0849	-0.156	0.177	0.016	1	0.899	1.011	0.856	1.194
Low	0[a]	.	.	.	.	.	.	1	.	.
**Self management/Compliance**										
High	0.102	0.1109	-0.115	0.320	0.854	1	0.355	1.108	0.891	1.377
Middle	0.141	0.0919	-0.039	0.322	2.369	1	0.124	1.152	0.962	1.379
Low	0[a]	.	.	.	.	.	.	1	.	.
**Academic behavior**										
High	-0.708	0.0926	-0.889	-0.526	58.366	1	0.000	0.493	0.411	0.591
Middle	-0.297	0.0704	-0.435	-0.159	17.792	1	0.000	0.743	0.647	0.853
Low	0[a]	.	.	.	.	.	.	1	.	.
**Antisocial Behavior**										
**Hostile/Irritable**										
High	-0.933	0.5267	-1.965	0.099	3.139	1	0.076	.393	0.140	1.104
Middle	0.384	0.1530	0.084	.684	6.298	1	0.012	1.468	1.088	1.981
Low	0[a]	.	.	.	.	.	.	1	.	.
**Anti-social/Aggressive**										
High	1.111	0.6183	-0.101	2.323	3.229	1	0.072	3.037	0.904	10.202
Middle	0.144	0.1752	-0.200	.487	0.671	1	0.413	1.154	0.819	1.627
Low	0[a]	.	.	.	.	.	.	1	.	.
**Defiant/Disruptive**										
High	1.969	0.5887	0.815	3.123	11.186	1	0.001	7.162	2.259	22.705
Middle	0.412	0.1656	0.088	0.737	6.192	1	0.013	1.510	1.091	2.089
Low	0[a]	.	.	.	.	.		1	.	.
(Scale)	1[b]									

a. Set to zero because this parameter is redundant.
b. Fixed at the displayed value.

C.3 Interpersonal sensitivity

Table S16. Goodness of Fit.

	Value	df	Value/df
Deviance	2904.017	4149	0.700
Scaled Deviance	2904.017	4149	
Pearson Chi-Square	5110.039	4149	1.232
Scaled Pearson Chi-Square	5110.039	4149	
Log Likelihoodb	-2156.481		
Akaike's Information Criterion (AIC)	4366.962		
Finite Sample Corrected AIC (AICC)	4367.124		
Bayesian Information Criterion (BIC)	4559.965		
Consistent AIC (CAIC)	4586.965		

Table S17. Omnibus Test.

Likelihood Ratio Chi-Square	df	Sig.
849.242	24	0.000

Table S18. Parameter Estimates.

Parameter		B	Std. Error	95% Wald Confidence Interval		Hypothesis Test			Exp(B)	95% Wald Confidence Interval for Exp(B)	
				Lower	Upper	Wald Chi-Square	df	Sig.		Lower	Upper
Threshold	[Interpersonal sensitivity level = Normal]	-0.284	0.0983	-0.477	-0.091	8.330	1	0.004	0.753	0.621	0.913
	[Interpersonal sensitivity level = Mild]	1.584	0.1048	1.379	1.790	228.525	1	0.000	4.875	3.970	5.987
	[Interpersonal sensitivity level = Moderate]	3.306	0.1448	3.022	3.590	521.050	1	0.000	27.269	20.530	36.219
**Father's educational level**											
Graduate and above		-0.076	0.2767	-0.618	0.466	0.076	1	0.783	0.927	0.539	1.594
University		-0.339	0.1237	-0.581	-0.096	7.504	1	0.006	0.713	0.559	0.908
High school		-0.275	0.0953	-0.461	-0.088	8.303	1	0.004	0.760	0.631	0.916
Junior high school		-0.268	0.0854	-0.435	-0.101	9.855	1	0.002	0.765	0.647	0.904
Elementary school and below		0[a]	.	.	.	.	.	.	1	.	.
**Mother's educational level**											
Graduate and above		-0.758	0.2952	-1.337	-0.180	6.601	1	0.010	0.468	0.263	0.835
University		-0.537	0.1242	-0.780	-0.293	18.681	1	0.000	0.585	0.458	0.746
High school		-0.315	0.0864	-0.484	-0.145	13.270	1	0.000	0.730	0.616	0.865
Junior high school		-0.145	0.0712	-0.284	-0.005	4.137	1	0.042	0.865	0.753	0.995
Elementary school and below		0[a]	.	.	.	.	.	.	1	.	.
**PA**											
≥7 time pw		-0.351	0.1311	-0.608	-0.094	7.151	1	0.007	0.704	0.545	0.911
5-6 time pw		-0.804	0.1182	-1.035	-0.572	46.279	1	0.000	0.448	0.355	0.564
3-4 time pw		-0.771	0.0843	-0.936	-0.606	83.636	1	0.000	0.463	0.392	0.546
1-2 time pw		-0.592	0.0616	-0.713	-0.472	92.344	1	0.000	0.553	0.490	0.624
0 time pw		0[a]	.	.	.	.	.	.	1	.	.
**Social Competence**											
**Peer relations**											
High		-0.548	.1105	-0.765	-0.331	24.590	1	0.000	0.578	0.466	0.718
Middle		-0.143	0.0883	-0.316	0.030	2.636	1	0.104	0.867	0.729	1.030

Low	0[a]	.	.	.	.	.	.	1	.	.
**Self management/Compliance**										
High	-0.108	0.1180	-0.339	0.123	0.839	1	0.360	0.898	0.712	1.131
Middle	0.022	0.0948	-0.164	0.208	0.054	1	0.817	1.022	0.849	1.231
Low	0[a]	.	.	.	.	.	.	1	.	.
**Academic behavior**										
High	-0.808	0.1025	-1.009	-0.608	62.257	1	0.000	0.446	0.364	0.545
Middle	-0.353	0.0744	-0.498	-0.207	22.487	1	0.000	0.703	0.608	0.813
Low	0[a]	.	.	.	.	.	.	1	.	.
**Antisocial Behavior**										
**Hostile/Irritable**										
High	-0.861	0.5573	-1.954	0.231	2.390	1	0.122	0.423	0.142	1.260
Middle	0.508	0.1561	0.202	0.814	10.580	1	0.001	1.662	1.224	2.256
Low	0[a]	.	.	.	.	.	.	1	.	.
**Anti-social/Aggressive**										
High	1.864	0.6203	0.648	3.080	9.032	1	0.003	6.450	1.912	21.753
Middle	0.126	0.1785	-0.224	0.476	0.500	1	0.479	1.135	0.800	1.610
Low	0[a]	.	.	.	.	.	.	1	.	.
**Defiant/Disruptive**										
High	1.322	0.5791	0.186	2.457	5.207	1	0.022	3.749	1.205	11.665
Middle	0.470	0.1677	0.141	0.798	7.839	1	0.005	1.599	1.151	2.222
Low	0[a]	.	.	.	.	.	.	1	.	.
(Scale)	1[b]									

a. Set to zero because this parameter is redundant.
b. Fixed at the displayed value.

C.4 Depression

Table S19. Goodness of Fit.

	Value	df	Value/df
Deviance	2902.528	4149	0.700
Scaled Deviance	2902.528	4149	
Pearson Chi-Square	4801.193	4149	1.157
Scaled Pearson Chi-Square	4801.193	4149	
Log Likelihoodb	-2110.281		
Akaike's Information Criterion (AIC)	4274.562		
Finite Sample Corrected AIC (AICC)	4274.724		
Bayesian Information Criterion (BIC)	4467.565		
Consistent AIC (CAIC)	4494.565		

Table S20. Omnibus Test.

Likelihood Ratio Chi-Square	df	Sig.
873.731	24	0.000

Table S21. Parameter Estimates.

Parameter		B	Std. Error	95% Wald Confidence Interval		Hypothesis Test			Exp(B)	95% Wald Confidence Interval for Exp(B)	
				Lower	Upper	Wald Chi-Square	df	Sig.		Lower	Upper
Threshold	[Depression level = Normal]	-0.314	0.0999	-0.509	-0.118	9.863	1	0.002	0.731	0.601	0.889
	[Depression level = Mild]	1.409	0.1057	1.202	1.616	177.818	1	0.000	4.091	3.326	5.033
	[Depression level = Moderate]	3.029	0.1404	2.753	3.304	465.164	1	0.000	20.670	15.697	27.219
**Father's educational level**											
Graduate and above		-0.256	0.2950	-0.834	0.322	0.751	1	0.386	0.774	0.434	1.381
University		-0.272	0.1284	-0.524	-0.020	4.487	1	0.034	0.762	0.592	0.980
High school		-0.240	0.0985	-0.433	-0.047	5.929	1	0.015	0.787	0.649	0.954
Junior high school		-0.281	0.0884	-0.454	-0.108	10.120	1	0.001	0.755	0.635	0.898
Elementary school and below		0[a]	.	.	.	.	.	.	1	.	.
**Mother's educational level**											
Graduate and above		-0.816	0.3115	-1.426	-0.205	6.861	1	0.009	0.442	0.240	0.814
University		-0.529	0.1283	-0.781	-0.278	17.018	1	0.000	0.589	0.458	0.757
High school		-0.465	0.0907	-0.642	-0.287	26.217	1	0.000	0.628	0.526	0.751
Junior high school		-0.189	0.0739	-0.334	-0.044	6.526	1	0.011	0.828	0.716	0.957
Elementary school and below		0[a]	.	.	.	.	.	.	1	.	.
**PA**											
≥7 time pw		-0.355	0.1352	-0.620	-0.090	6.886	1	0.009	0.701	0.538	0.914
5-6 time pw		-0.918	0.1264	-1.166	-0.670	52.738	1	0.000	0.399	0.312	0.512
3-4 time pw		-0.818	0.0881	-0.991	-0.646	86.232	1	0.000	0.441	0.371	0.524
1-2 time pw		-0.652	0.0640	-0.777	-0.526	103.575	1	0.000	0.521	0.460	0.591
0 time pw		0[a]	.	.	.	.	.	.	1	.	.
**Social Competence**											
**Peer relations**											
High		-0.437	0.1154	-0.663	-0.211	14.350	1	0.000	0.646	0.515	0.810
Middle		-0.089	0.0911	-0.267	0.090	0.947	1	0.331	0.915	0.766	1.094
Low		0[a]	.	.	.	.	.	.	1	.	.

**Self management/Compliance**										
High	-0.452	0.1218	-0.690	-0.213	13.746	1	0.000	0.637	0.501	0.808
Middle	-0.224	0.0963	-0.413	-0.035	5.414	1	0.020	0.799	0.662	0.965
Low	0[a]	.	.	.	.	.	.	1	.	.
**Academic behavior**										
High	-0.653	0.1074	-0.864	-0.443	37.043	1	0.000	0.520	0.422	0.642
Middle	-0.306	0.0772	-0.457	-0.155	15.701	1	0.000	0.736	0.633	0.857
Low	0[a]	.	.	.	.	.	.	1	.	.
**Antisocial Behavior**										
**Hostile/Irritable**										
High	-0.391	0.5291	-1.428	0.646	0.547	1	0.460	0.676	0.240	1.907
Middle	0.710	0.1548	0.407	1.014	21.060	1	0.000	2.035	1.502	2.756
Low	0[a]	.	.	.	.	.	.	1	.	.
**Anti-social/Aggressive**										
High	0.665	0.6259	-0.562	1.892	1.129	1	0.288	1.945	0.570	6.631
Middle	0.102	0.1798	-0.251	0.454	0.319	1	0.572	1.107	0.778	1.575
Low	0[a]	.	.	.	.	.	.	1	.	.
**Defiant/Disruptive**										
High	2.086	.5942	0.922	3.251	12.327	1	0.000	8.055	2.513	25.814
Middle	0.690	0.1668	0.363	1.017	17.137	1	0.000	1.994	1.438	2.765
Low	0[a]	.	.	.	.	.	.	1	.	.
(Scale)	1[b]									

a. Set to zero because this parameter is redundant.

b. Fixed at the displayed value.

C.5 Anxiety

Table S22. Goodness of Fit.

	Value	df	Value/df
Deviance	2720.719	4149	0.656
Scaled Deviance	2720.719	4149	
Pearson Chi-Square	4770.272	4149	1.150
Scaled Pearson Chi-Square	4770.272	4149	
Log Likelihoodb	-1970.160		
Akaike's Information Criterion (AIC)	3994.320		
Finite Sample Corrected AIC (AICC)	3994.481		
Bayesian Information Criterion (BIC)	4187.322		
Consistent AIC (CAIC)	4214.322		

Table S23. Omnibus Test.

Likelihood Ratio Chi-Square	df	Sig.
726.188	24	0.000

Table S24. Parameter Estimates.

Parameter		B	Std. Error	95% Wald Confidence Interval		Hypothesis Test			Exp(B)	95% Wald Confidence Interval for Exp(B)	
				Lower	Upper	Wald Chi-Square	df	Sig.		Lower	Upper
Threshold	[Anxiety level = Normal]	0.057	0.1036	-0.146	0.260	0.301	1	0.583	1.059	0.864	1.297
	[Anxiety level = Mild]	1.819	0.1120	1.599	2.038	263.676	1	0.000	6.165	4.950	7.679
	[Anxiety level = Moderate]	3.344	0.1490	3.052	3.636	503.448	1	0.000	28.326	21.151	37.935
**Father's educational level**											
Graduate and above		-0.192	0.3028	-0.786	0.401	0.404	1	.525	0.825	0.456	1.493
University		-0.339	0.1358	-0.605	-0.072	6.217	1	0.013	0.713	0.546	0.930
High school		-0.213	0.1031	-0.415	-0.011	4.273	1	0.039	0.808	0.660	0.989
Junior high school		-0.260	0.0930	-0.442	-0.078	7.814	1	0.005	0.771	0.643	0.925
Elementary school and below		0[a]	.	.	.	.	.	.	1	.	.
**Mother's educational level**											
Graduate and above		-0.626	0.3179	-1.249	-0.003	3.875	1	0.049	0.535	0.287	0.997
University		-0.538	0.1381	-0.808	-0.267	15.154	1	0.000	0.584	0.446	0.766
High school		-0.249	0.0938	-0.433	-0.065	7.058	1	0.008	0.779	0.648	0.937
Junior high school		-0.161	0.0783	-0.314	-0.007	4.201	1	0.040	0.852	0.730	0.993
Elementary school and below		0[a]	.	.	.	.	.	.	1	.	.
**PA**											
≥7 time pw		-0.404	0.1473	-0.693	-0.116	7.536	1	0.006	0.667	0.500	0.891
5-6 time pw		-0.718	0.1300	-0.973	-0.464	30.537	1	0.000	0.488	0.378	0.629
3-4 time pw		-0.607	0.0909	-0.785	-0.429	44.524	1	0.000	0.545	0.456	0.651
1-2 time pw		-0.542	0.0678	-0.675	-0.409	64.029	1	0.000	0.581	0.509	0.664
0 time pw		0[a]	.	.	.	.	.	.	1	.	.
**Social Competence**											
**Peer relations**											
High		-0.544	0.1208	-0.781	-0.308	20.306	1	0.000	0.580	0.458	0.735
Middle		-0.133	0.0943	-0.318	0.051	2.002	1	0.157	0.875	0.727	1.053
Low		0[a]	.	.	.	.	.	.	1	.	.
**Self management/Compliance**											

High	-0.373	.1272	-0.622	-0.124	8.604	1	0.003	0.689	0.537	0.884
Middle	-0.194	0.0999	-0.390	0.002	3.762	1	0.052	0.824	0.677	1.002
Low	0[a]	.	.	.	.	.	.	1	.	.
**Academic behavior**										
High	-0.589	0.1132	-0.811	-0.367	27.118	1	0.000	0.555	0.444	0.692
Middle	-0.249	0.0808	-0.407	-0.091	9.499	1	0.002	0.780	0.665	0.913
Low	0[a]	.	.	.	.	.	.	1	.	.
**Antisocial Behavior**										
**Hostile/Irritable**										
High	-0.357	0.5429	-1.421	0.707	0.432	1	0.511	0.700	0.241	2.028
Middle	.811	0.1561	.505	1.117	27.003	1	0.000	2.250	1.657	3.055
Low	0[a]	.	.	.	.	.	.	1	.	.
**Anti-social/Aggressive**										
High	1.125	0.6241	-0.098	2.348	3.251	1	0.071	3.081	0.907	10.469
Middle	.241	0.1812	-0.115	0.596	1.763	1	.184	1.272	0.892	1.814
Low	0[a]	.	.	.	.	.	.	1	.	.
**Defiant/Disruptive**										
High	1.832	0.5889	0.678	2.986	9.678	1	0.002	6.247	1.970	19.815
Middle	0.488	0.1713	0.153	0.824	8.133	1	0.004	1.630	1.165	2.280
Low	0[a]	.	.	.	.	.	.	1	.	.
(Scale)	1[b]									

a. Set to zero because this parameter is redundant.
b. Fixed at the displayed value.

C.6 Hostility

Table S25. Goodness of Fit.

	Value	df	Value/df
Deviance	2550.470	4149	0.615
Scaled Deviance	2550.470	4149	
Pearson Chi-Square	4883.299	4149	1.177
Scaled Pearson Chi-Square	4883.299	4149	
Log Likelihoodb	-1808.160		
Akaike's Information Criterion (AIC)	3670.320		
Finite Sample Corrected AIC (AICC)	3670.481		
Bayesian Information Criterion (BIC)	3863.323		
Consistent AIC (CAIC)	3890.323		

Table S26. Omnibus Test.

Likelihood Ratio Chi-Square	df	Sig.
703.359	24	0.000

Table S27. Parameter Estimates.

Parameter		B	Std. Error	95% Wald Confidence Interval		Hypothesis Test			Exp(B)	95% Wald Confidence Interval for Exp(B)	
				Lower	Upper	Wald Chi-Square	df	Sig.		Lower	Upper
Threshold	[Hostility level = Normal]	0.249	0.1092	0.035	0.463	5.204	1	0.023	1.283	1.036	1.589
	[Hostility level = Mild]	1.930	0.1189	1.697	2.163	263.431	1	0.000	6.887	5.455	8.694
	[Hostility level = Moderate]	3.342	0.1545	3.039	3.645	467.631	1	0.000	28.271	20.883	38.272
**Father's educational level**											
Graduate and above		-0.389	0.3262	-1.028	0.250	1.422	1	0.233	0.678	0.358	1.284
University		-0.162	0.1453	-0.447	0.123	1.242	1	0.265	0.850	0.640	1.131
High school		-0.256	0.1126	-0.477	-0.036	5.188	1	0.023	0.774	0.621	0.965
Junior high school		-0.253	0.1011	-0.451	-0.055	6.272	1	0.012	0.776	0.637	0.946
Elementary school and below		0[a]	.	.	.	.	.	.	1	.	.
**Mother's educational level**											
Graduate and above		-0.313	0.3267	-0.953	0.327	0.920	1	0.338	0.731	0.385	1.387
University		-0.441	0.1478	-0.731	-0.152	8.920	1	0.003	0.643	0.481	0.859
High school		-0.303	0.1036	-0.506	-0.100	8.540	1	0.003	0.739	0.603	0.905
Junior high school		-0.156	0.0861	-0.325	0.012	3.299	1	0.069	0.855	0.723	1.012
Elementary school and below		0[a]	.	.	.	.	.	.	1	.	.
**PA**											
≥7 time pw		-0.326	0.1600	-0.639	-0.012	4.146	1	0.042	0.722	0.528	0.988
5-6 time pw		-0.613	0.1408	-0.889	-0.337	18.966	1	0.000	0.542	0.411	0.714
3-4 time pw		-0.507	0.0992	-0.701	-0.312	26.097	1	0.000	0.602	0.496	0.732
1-2 time pw		-0.484	0.0743	-0.630	-0.339	42.492	1	0.000	0.616	0.533	0.713
0 time pw		0[a]	.	.	.	.	.	.	1	.	.
**Social Competence**											
**Peer relations**											
High		-0.566	0.1305	-0.822	-0.310	18.804	1	0.000	0.568	0.440	0.733
Middle		-0.267	0.0992	-0.461	-0.072	7.229	1	0.007	0.766	0.631	0.930
Low		0[a]	.	.	.	.	.	.	1	.	.

**Self management/Compliance**										
High	-0.541	0.1380	-0.812	-0.271	15.390	1	0.000	0.582	0.444	0.763
Middle	-0.179	0.1044	-0.384	0.025	2.946	1	0.086	0.836	0.681	1.026
Low	0[a]	.	.	.	.	.	.	1	.	.
**Academic behavior**										
High	-0.599	0.1247	-0.843	-0.354	23.058	1	0.000	0.550	0.430	0.702
Middle	-0.325	0.0862	-0.494	-0.156	14.230	1	0.000	0.722	0.610	0.855
Low	0[a]	.	.	.	.	.	.	1	.	.
**Antisocial Behavior**										
**Hostile/Irritable**										
High	-0.203	0.5427	-1.267	0.861	0.140	1	0.708	0.816	0.282	2.364
Middle	0.715	0.1618	0.398	1.032	19.506	1	0.000	2.044	1.488	2.806
Low	0[a]	.	.	.	.	.	.	1	.	.
**Anti-social/Aggressive**										
High	1.681	0.6175	0.471	2.891	7.409	1	0.006	5.370	1.601	18.014
Middle	0.401	0.1828	0.043	0.759	4.813	1	0.028	1.493	1.044	2.137
Low	0[a]	.	.	.	.	.	.	1	.	.
**Defiant/Disruptive**										
High	1.511	0.5779	0.378	2.644	6.837	1	0.009	4.531	1.460	14.063
Middle	0.653	0.1726	0.314	0.991	14.296	1	0.000	1.921	1.369	2.694
Low	0[a]	.	.	.	.	.	.	1	.	.
(Scale)	1[b]									

a. Set to zero because this parameter is redundant.
b. Fixed at the displayed value.

C.7 Phobic anxiety

Table S28. Goodness of Fit.

	Value	df	Value/df
Deviance	2406.235	4149	0.580
Scaled Deviance	2406.235	4149	
Pearson Chi-Square	4516.522	4149	1.089
Scaled Pearson Chi-Square	4516.522	4149	
Log Likelihoodb	-1725.344		
Akaike's Information Criterion (AIC)	3504.688		
Finite Sample Corrected AIC (AICC)	3504.849		
Bayesian Information Criterion (BIC)	3697.691		
Consistent AIC (CAIC)	3724.691		

Table S29. Omnibus Test.

Likelihood Ratio Chi-Square	df	Sig.
581.416	24	0.000

Table S30. Parameter Estimates.

Parameter		B	Std. Error	95% Wald Confidence Interval		Hypothesis Test			Exp(B)	95% Wald Confidence Interval for Exp(B)	
				Lower	Upper	Wald Chi-Square	df	Sig.		Lower	Upper
Threshold	[Phobic anxiety level = Normal]	0.450	0.1112	0.232	0.668	16.391	1	0.000	1.568	1.261	1.950
	[Phobic anxiety level = Mild]	2.163	0.1217	1.924	2.401	315.757	1	0.000	8.696	6.851	11.039
	[Phobic anxiety level = Moderate]	3.877	0.1735	3.537	4.217	499.372	1	0.000	48.278	34.362	67.831
**Father's educational level**											
Graduate and above		-0.288	.3281	-0.931	0.355	0.769	1	0.381	0.750	0.394	1.427
University		-0.295	.1467	-0.583	-0.007	4.041	1	0.044	0.745	0.558	0.993
High school		-0.270	.1123	-0.490	-0.050	5.782	1	0.016	0.763	0.613	0.951
Junior high school		-0.224	.1005	-0.421	-0.027	4.948	1	0.026	0.800	0.657	0.974
Elementary school and below		0[a]	.	.	.	.	.	.	1	.	.
**Mother's educational level**											
Graduate and above		-0.457	.3384	-1.120	0.206	1.824	1	0.177	0.633	0.326	1.229
University		-0.418	.1491	-0.710	-0.126	7.876	1	0.005	0.658	0.491	0.881
High school		-0.232	.1029	-0.433	-0.030	5.065	1	0.024	0.793	0.648	0.971
Junior high school		-0.090	0.0852	-0.257	0.076	1.128	1	0.288	0.914	0.773	1.079
Elementary school and below		0[a]	.	.	.	.	.	.	1	.	.
**PA**											
≥7 time pw		-0.363	.1577	-0.672	-0.054	5.287	1	0.021	0.696	0.511	0.948
5-6 time pw		-0.610	.1379	-0.880	-0.340	19.588	1	0.000	0.543	0.415	0.712
3-4 time pw		-0.683	.1013	-0.882	-0.485	45.477	1	0.000	0.505	0.414	0.616
1-2 time pw		-0.520	0.0733	-0.664	-0.377	50.452	1	0.000	0.594	0.515	0.686
0 time pw		0[a]	.	.	.	.	.	.	1	.	.
**Social Competence**											
**Peer relations**											
High		-0.493	0.1311	-0.750	-0.236	14.136	1	0.000	0.611	0.472	0.790
Middle		-0.133	0.1015	-0.332	0.066	1.728	1	0.189	0.875	0.717	1.068

Low	0[a]	.	.	.	.	.	.	1	.	.
**Self management/Compliance**										
High	-0.221	0.1381	-0.492	0.049	2.573	1	0.109	0.801	0.611	1.050
Middle	-0.087	0.1076	-0.298	.124	0.649	1	0.420	0.917	0.743	1.132
Low	0[a]	.	.	.	.	.	.	1	.	.
**Academic behavior**										
High	-0.656	0.1228	-0.896	-0.415	28.495	1	0.000	0.519	0.408	0.660
Middle	-0.346	0.0871	-0.516	-0.175	15.774	1	0.000	0.708	0.597	0.839
Low	0[a]	.	.	.	.	.	.	1	.	.
**Antisocial Behavior**										
**Hostile/Irritable**										
High	-0.168	0.5402	-1.227	0.890	0.097	1	0.755	0.845	0.293	2.436
Middle	0.892	0.1619	0.574	1.209	30.342	1	0.000	2.439	1.776	3.350
Low	0[a]	.	.	.	.	.	.	1	.	.
**Anti-social/Aggressive**										
High	1.461	0.6207	0.244	2.678	5.538	1	0.019	4.310	1.277	14.549
Middle	0.215	0.1902	-0.158	.587	1.273	1	0.259	1.239	0.854	1.799
Low	0[a]	.	.	.	.	.	.	1	.	.
**Defiant/Disruptive**										
High	1.156	0.5886	0.002	2.309	3.855	1	0.050	3.176	1.002	10.069
Middle	0.668	0.1767	0.321	1.014	14.281	1	0.000	1.950	1.379	2.757
Low	0[a]	.	.	.	.	.	.	1	.	.
(Scale)	1[b]									

a. Set to zero because this parameter is redundant.
b. Fixed at the displayed value.

C.8 Paranoid ideation

Table S31. Goodness of Fit.

	Value	df	Value/df
Deviance	2477.651	4149	0.597
Scaled Deviance	2477.651	4149	
Pearson Chi-Square	4459.028	4149	1.075
Scaled Pearson Chi-Square	4459.028	4149	
Log Likelihoodb	-1784.155		
Akaike's Information Criterion (AIC)	3622.309		
Finite Sample Corrected AIC (AICC)	3622.471		
Bayesian Information Criterion (BIC)	3815.312		
Consistent AIC (CAIC)	3842.312		

Table S32. Omnibus Test.

Likelihood Ratio Chi-Square	df	Sig.
739.229	24	0.000

Table S33 Parameter Estimates.

Parameter		B	Std. Error	95% Wald Confidence Interval		Hypothesis Test			Exp(B)	95% Wald Confidence Interval for Exp(B)	
				Lower	Upper	Wald Chi-Square	df	Sig.		Lower	Upper
Threshold	[Paranoid ideation level = Normal]	0.130	0.1076	-0.081	0.341	1.453	1	0.228	1.139	0.922	1.406
	[Paranoid ideation level = Mild]	1.999	0.1190	1.766	2.233	282.302	1	0.000	7.385	5.849	9.325
	[Paranoid ideation level = Moderate]	3.705	0.1729	3.367	4.044	459.518	1	0.000	40.667	28.981	57.066
**Father's educational level**											
Graduate and above		0.149	0.3005	-0.440	0.738	0.245	1	0.620	1.161	0.644	2.091
University		-0.194	0.1421	-0.472	0.085	1.858	1	0.173	0.824	0.624	1.089
High school		-0.288	0.1105	-0.505	-0.071	6.792	1	0.009	0.750	0.604	0.931
Junior high school		-0.275	0.0993	-0.470	-0.081	7.699	1	0.006	0.759	0.625	0.922
Elementary school and below		0[a]	.	.	.	.	.	.	1	.	.
**Mother's educational level**											
Graduate and above		-0.707	0.3239	-1.342	-0.073	4.770	1	0.029	0.493	0.261	0.930
University		-0.439	0.1447	-0.723	-0.156	9.221	1	0.002	0.644	0.485	0.856
High school		-0.209	0.1009	-0.407	-0.012	4.306	1	0.038	0.811	0.666	0.988
Junior high school		-0.152	0.0847	-0.318	0.014	3.230	1	0.072	0.859	0.727	1.014
Elementary school and below		0[a]	.	.	.	.	.	.	1	.	.
**PA**											
≥7 time pw		-0.338	0.1522	-0.636	-0.039	4.916	1	0.027	0.714	0.529	0.962
5-6 time pw		-0.753	0.1386	-1.025	-0.481	29.498	1	0.000	0.471	0.359	0.618
3-4 time pw		-0.609	0.0963	-0.798	-0.420	39.927	1	0.000	0.544	0.450	0.657
1-2 time pw		-0.642	0.0726	-0.784	-0.500	78.281	1	0.000	0.526	0.457	0.607
0 time pw		0[a]	.	.	.	.	.	.	1	.	.
**Social Competence**											
**Peer relations**											
High		-0.710	0.1280	-0.961	-0.459	30.747	1	0.000	0.492	0.383	0.632
Middle		-0.323	0.0979	-0.515	-0.131	10.890	1	0.001	0.724	0.598	0.877

Low	0[a]	.	.	.	.	.	.	1	.	.
**Self management/Compliance**										
High	-0.371	0.1350	-0.636	-0.106	7.552	1	0.006	0.690	0.530	0.899
Middle	-0.157	0.1036	-0.360	0.046	2.296	1	0.130	0.855	0.698	1.047
Low	0a	.	.	.	.	.	.	1	.	.
**Academic behavior**										
High	-0.540	0.1217	-0.779	-0.302	19.701	1	0.000	0.583	0.459	0.740
Middle	-0.280	0.0855	-0.447	-0.112	10.698	1	0.001	0.756	0.639	0.894
Low	0[a]	.	.	.	.	.	.	1	.	.
**Antisocial Behavior**										
**Hostile/Irritable**										
High	0.053	0.5153	-0.957	1.063	0.011	1	0.918	1.054	0.384	2.895
Middle	0.978	0.1571	0.670	1.286	38.779	1	0.000	2.660	1.955	3.619
Low	0[a]	.	.	.	.	.	.	1	.	.
**Anti-social/Aggressive**										
High	1.067	0.6136	-0.136	2.269	3.022	1	0.082	2.906	0.873	9.673
Middle	0.143	0.1849	-0.219	0.506	0.602	1	0.438	1.154	0.803	1.658
Low	0[a]	.	.	.	.	.	.	1	.	.
**Defiant/Disruptive**										
High	1.802	0.5826	0.660	2.943	9.565	1	0.002	6.060	1.935	18.980
Middle	0.677	0.1718	0.341	1.014	15.542	1	0.000	1.969	1.406	2.757
Low	0[a]	.	.	.	.	.	.	1	.	.
(Scale)	1[b]									

a. Set to zero because this parameter is redundant.
b. Fixed at the displayed value.

C.9 Psychoticism

Table S34. Goodness of Fit.

	Value	df	Value/df
Deviance	2488.330	4149	0.600
Scaled Deviance	2488.330	4149	
Pearson Chi-Square	4697.341	4149	1.132
Scaled Pearson Chi-Square	4697.341	4149	
Log Likelihoodb	-1749.538		
Akaike's Information Criterion (AIC)	3553.075		
Finite Sample Corrected AIC (AICC)	3553.237		
Bayesian Information Criterion (BIC)	3746.078		
Consistent AIC (CAIC)	3773.078		

Table S35. Omnibus Test.

Likelihood Ratio Chi-Square	df	Sig.
779.436	24	0.000

Table S36. Parameter Estimates.

Parameter		B	Std. Error	95% Wald Confidence Interval		Hypothesis Test			Exp(B)	95% Wald Confidence Interval for Exp(B)	
				Lower	Upper	Wald Chi-Square	df	Sig.		Lower	Upper
Threshold	[Psychoticism level = Normal]	0.048	0.1078	-0.163	0.259	0.201	1	0.654	1.049	0.850	1.296
	[Psychoticism level = Mild]	1.993	0.1203	1.757	2.229	274.293	1	0.000	7.337	5.796	9.289
	[Psychoticism level = Moderate]	3.761	0.1833	3.402	4.120	420.907	1	0.000	42.990	30.014	61.575
**Father's educational level**											
Graduate and above		-0.115	0.3193	-0.740	0.511	0.129	1	.720	0.892	0.477	1.667
University		-0.306	0.1452	-0.591	-0.022	4.449	1	0.035	0.736	0.554	0.979
High school		-0.357	0.1110	-0.574	-0.139	10.313	1	0.001	0.700	0.563	0.870
Junior high school		-0.351	0.0996	-0.546	-0.156	12.423	1	0.000	0.704	0.579	0.856
Elementary school and below		0[a]	.	.	.	.	.	.	1	.	.
**Mother's educational level**											
Graduate and above		-0.724	0.3417	-1.394	-0.054	4.491	1	0.034	0.485	0.248	0.947
University		-0.514	0.1512	-0.810	-0.218	11.565	1	0.001	0.598	0.445	0.804
High school		-0.186	0.1025	-0.387	0.015	3.298	1	0.069	0.830	0.679	1.015
Junior high school		-0.102	0.0859	-0.270	0.066	1.415	1	0.234	0.903	0.763	1.068
Elementary school and below		0[a]	.	.	.	.	.	.	1	.	.
**PA**											
≥7 time pw		-0.482	0.1603	-0.797	-0.168	9.058	1	0.003	0.617	0.451	0.845
5-6 time pw		-0.803	0.1424	-1.082	-0.524	31.799	1	0.000	0.448	0.339	0.592
3-4 time pw		-0.671	0.0986	-0.864	-0.477	46.206	1	0.000	0.511	0.422	0.621
1-2 time pw		-0.670	0.0735	-0.814	-0.526	83.147	1	0.000	0.512	0.443	0.591
0 time pw		0[a]	.	.	.	.	.	.	1	.	.
**Social Competence**											
**Peer relations**											
High		-0.820	0.1306	-1.076	-0.563	39.355	1	0.000	0.441	0.341	0.569
Middle		-0.312	0.0986	-0.505	-0.119	10.004	1	0.002	0.732	0.603	0.888

Low	0[a]	.	.	.	.	.	.	1	.	.
**Self management/Compliance**										
High	-0.420	0.1365	-0.688	-0.152	9.459	1	0.002	0.657	0.503	0.859
Middle	-0.227	0.1042	-0.431	-0.022	4.731	1	0.030	0.797	0.650	0.978
Low	0[a]	.	.	.	.	.	.	1	.	.
**Academic behavior**										
High	-0.469	0.1243	-0.713	-0.226	14.273	1	0.000	0.625	0.490	0.798
Middle	-0.243	0.0866	-0.413	-0.074	7.892	1	0.005	0.784	0.662	0.929
Low	0[a]	.	.	.	.	.	.	1	.	.
**Antisocial Behavior**										
**Hostile/Irritable**										
High	0.548	0.4825	-0.398	1.494	1.290	1	0.256	1.730	0.672	4.453
Middle	0.790	0.1604	0.476	1.105	24.267	1	0.000	2.204	1.609	3.018
Low	0[a]	.	.	.	.	.	.	1	.	.
**Anti-social/Aggressive**										
High	0.469	0.6215	-0.749	1.687	0.569	1	0.450	1.598	0.473	5.404
Middle	0.267	0.1843	-0.094	0.628	2.098	1	0.148	1.306	0.910	1.874
Low	0[a]	.	.	.	.	.	.	1	.	.
**Defiant/Disruptive**										
High	1.423	0.5941	0.259	2.587	5.737	1	0.017	4.149	1.295	13.292
Middle	0.780	0.1716	0.443	1.116	20.651	1	0.000	2.181	1.558	3.052
Low	0[a]	.	.	.	.	.	.	1	.	.
(Scale)	1[b]									

a. Set to zero because this parameter is redundant.
b. Fixed at the displayed value.
